# Synthetic biology of oncolytic bacteria: Comparative microbial chassis and precise killing strategies

**DOI:** 10.1016/j.toxrep.2026.102292

**Published:** 2026-06-11

**Authors:** Mustafa Attiyah Hadid, Nawfal Haitham Al Shaikhli, Omar Khalid Suhail, Samar T. Hameed, Muhtada Ali Challoob

**Affiliations:** a Applied Pathological Analysis, College of Science, Al-Nahrain University, Baghdad, Iraq; b College of Pharmacy, Al-Nahrain University, 60th Street, Northern Complex, Al-Kadhimiya, Baghdad 10006, Iraq; c College of Pharmacy, University of Misan, Maysan, Iraq

**Keywords:** Cytotoxic payloads, Gene circuit, Microbial Cancer Therapy, Synthetic biology, Tumor targeting

## Abstract

The field of synthetic biology has become a revolutionary tool for engineering microorganisms capable of precise, programmed cancer treatment. Unlike conventional cancer treatments, which lack safe, selective toxicity, engineered microbial cells can detect tumor-specific signals, target hypoxic environments, and deliver cytotoxic payloads more effectively in both space and time. This review presents the most recent advancements in microbial chassis engineering, including *Escherichia coli*, *Salmonella*, *Clostridium*, *Bifidobacterium*, *Vibrio cholera*, *Shigella* species and *L. monocytogenes* along with their potential uses in targeted cancer therapy through toxin delivery, prodrug conversion, immune modulation, and tumor-specific surface display. We discuss key synthetic biology techniques that enhance safety, specificity, and genetic stability, including clustered regularly interspaced short palindromic repeat-associated protein 9 (CRISPR/Cas9)-based genome editing, genetic logic circuits, and kill-switch systems. This review provides some highlights about the role of synthetic biology in developing oncolytic bacteria with precise targeting abilities and enhanced therapeutic stability. By analyzing comparative microbial chassis and the implementation of precise killing strategies, we address current clinical challenges and explore the future of oncolytic bacterial therapy.

## Highlights

• Engineered bacteria enable targeted cancer treatment.
• Synthetic biology promotes tumor-specific cytotoxicity.
• Microbial toxins provide strong anticancer pathways.
• Gene circuits enhance the safety and control of therapy.
• Tumor microenvironment promotes microbial targeting.

## Graphical Abstract

**Figure:**
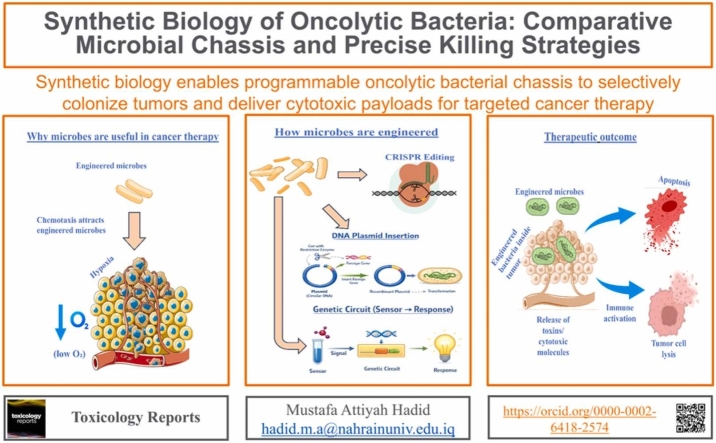

## 1 Introduction

Cancer represents one of the most substantial public health challenges worldwide. Each day, more than 52,900 individuals are diagnosed with cancer, and the disease accounts for over 27,000 deaths globally [1]. Malignant diseases still represent the second leading cause of mortality worldwide, despite extensive efforts to identify novel risk factors, develop early diagnostic biomarkers, and advance alternative therapeutic strategies [1]. It is predictable that by 2040, there will be 28 million new cancer cases and 16.2 million cancer-related deaths worldwide [2].

**Fig. 1:**
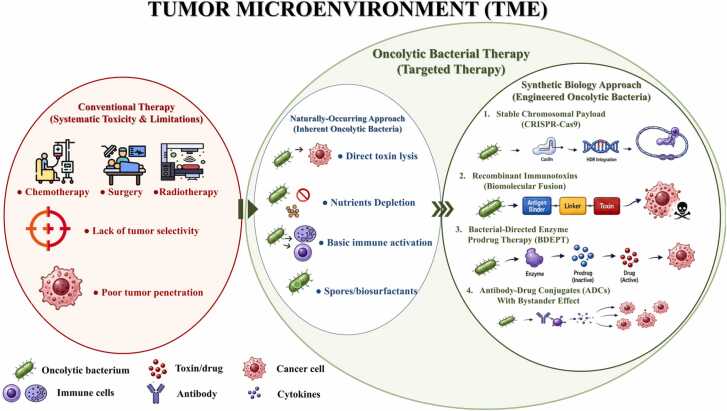
Schematic illustration of Conventional and Oncolytic Bacterial Therapeutic Strategies within the Tumor Microenvironment.

Many synthetic compounds and conventional chemotherapeutic agents for cancer treatment remain frequently poor in tumor selectivity. Thus, in addition to targeting cancer cells, hormonal chemotherapeutics and combination drug regimens also injure healthy cells and contribute to the development of drug resistance [3]. Furthermore, these cytotoxic agents are linked with potentially life-threatening side effects, which can often exceed the aggressiveness of the cancer itself [4] by accumulating through passive diffusion unlike living bacteria which can actively penetrate deep into tumors, bypassing aggregation near blood arteries [5], [6]. However, bacterial oncolytic therapy has gained a lot of attention in cancer treatment. This treatment targets and eliminates cancer cells by utilizing the particular features of specific bacteria (Fig. 1). These bacteria target hypoxic areas in solid tumors that are often resistant to traditional treatments [7]. They have the potential to serve as antitumor agents by multiple mechanisms, including the production of toxins, enzymes, and biosurfactants [8]. Therefore, one of the most promising alternative methods for treating cancer is bacterial oncolytic therapy. Numerous facultative or obligatory anaerobic bacteria, including *Salmonella* species, *Escherichia coli*, *Bifidobacterium*, *Listeria*, and *Clostridium*, have the ability to target and kill tumors. William B. Coley employed Coley's toxin and streptococcal cells to treat patients with terminal malignancies more than a century ago [9]. Bacteria can selectively proliferate and colonize tumors due to the tumor microenvironment's (TME) distinct features. For instance, *Salmonella* has been observed to localize to tumors at more than 10,000 times the density found in normal tissues [10]. Additionally, Live bacteria have distinct advantages over typical anticancer treatments by enhancing antitumor effects through innate tumor-targeting capabilities and potentially improving particular immune recognition [11]. However, balancing the need for bacteria to avoid host antimicrobial defenses while activating antitumor immunity within the tumor-microenvironment (TME) remains difficult. A recent study uncovered a novel hysteresis-based mechanism in which bacteria take advantage of preexisting interleukin−10 (IL−10) receptor expression across various cell types in the TME to shield tumor-associated macrophages (TAMs) from neutrophil phagocytosis while simultaneously activating and expanding exhausted tumor-resident CD8 + T cells [12]. Oncolytic bacteria have been shown in numerous trials to efficiently regress tumors; nevertheless, their clinical usage is complicated by issues such systemic toxicity, limited therapeutic efficacy, and unpredictable bacterial replication [13]. Moreover, microorganisms can be genetically altered to produce anticancer drugs, enhance the immune system, and improve their therapeutic properties. Numerous bacterial strains are currently undergoing clinical studies. The large taxonomic diversity of bacteria offers a great chance to discover new bacterial anticancer drugs, and the ease of genetic modification enhances the medicinal potential of bacteria [14].

While these natural bacterial characteristics provide a foundation for therapy, the transition to clinical efficacy requires the precise logic and stability offered by synthetic biology. Synthetic biology advancements allow for the rational design of improved oncolytic bacterial strains by attenuating virulence characteristics and adding customized therapeutic payloads, with some candidates now proceeding into clinical evaluation [15], [16]. Optimizing the spatiotemporal regulation of bacterial therapeutic effects is critical for maximizing drug accumulation, increasing resource efficiency, and minimizing damage to healthy tissues. For this reason, engineered oncolytic bacteria frequently use regulated gene expression systems, which incorporate specialized promoter elements, to enable for precise control of therapeutic payload delivery *in vivo* [17], [18]. Classical microbiology often takes advantage of native microbial features through strain screening [19], adaptive evolution [20], and either disease attenuation or probiotic optimization [21]. Synthetic biology, on the other hand, emphasizes rational and modular design, using programmable sensors, genetic circuits, and effectors to provide precise, tunable, multilayer control over bacterial behaviors and therapeutic outputs [22].

Therefore, this review aims to evaluate the modern synthetic biology landscape and its role in optimizing microbial chassis for oncolytic therapy. It provides an overview of microbial—tumor interactions and a comparative analysis of microbial chassis in terms of synthetic biology engineering mechanisms, antitumor and immunity activation mechanisms, and safety/spatiotemporal controls. Also, how these manipulated microbes deliver cytotoxic drugs to tumors, and the use of genome editing techniques like CRISPR/Cas9 and synthetic gene circuits to enhance safety and specificity. Additionally, the review evaluates the naturally-occurring physiological characteristics of various bacterial chassis and their innate ability to colonize and target the tumor microenvironment.

## 2 Historical background

There are well-established connections between the chronological study of microorganisms—mainly bacteria—and the evolving understanding of human diseases, including cancer [23]. Bacteria have been used to cure cancer since the nineteenth century. In 1867, German physician Wilhelm Busch documented a case of a cancer patient whose tumor growth reduced following a severe infection with erysipelas, today known as *Streptococcus pyogenes* [24]. In the early time of nineteenth-century, medical archives documented cases in which malignant tumors seemed to regress in patients suffering from concurrent bacterial infections [25]. In the late nineteenth century, William B. Coley observed head and neck cancer patients in significant remission after infections [24]. Based on some of the patients' records, he developed a "paradoxical" cancer treatment involving the intravenous infusion of heat-inactivated *Serratia pyogenes* and *Serratia marcescens*, also known as Coley's toxin.

During the next 40 years, a variety of sick persons experienced varying degrees of remission from sarcoma using "Coley's toxin," with 10-year survival rates comparable to those of people receiving modern conventional treatment. Coley's accomplishment earned him the label "the father of cancer immunotherapy." His findings inspired research into bacterial-based cancer therapies, ushering in a new age of cancer treatment [26]. Within this framework, some of the earliest anticancer “vaccines” were developed, based on attenuated or inactivated microorganisms. Nonetheless, the therapeutic results were inconsistent and difficult to reproduce, and the efficacy of these vaccines was not consistent worldwide; a formulation that showed promise against one type of cancer often proved ineffective against others. Consequently, by the mid-twentieth century, the bacterial concept (or parasitic) oncogenesis had mainly fallen out of favor among scientific and medical institutions [23]. However, the discovery of *Helicobacter pylori* which established the link between bacterial infection and gastric malignancy in the late twentieth century reversed this dogmatic disapproval, reviving interest in the topic and opening the route for contemporary studies into exact bacterial-cancer molecular interactions [27].

## 3 Factors driving bacterial selective tumor colonization

**Fig. 2:**
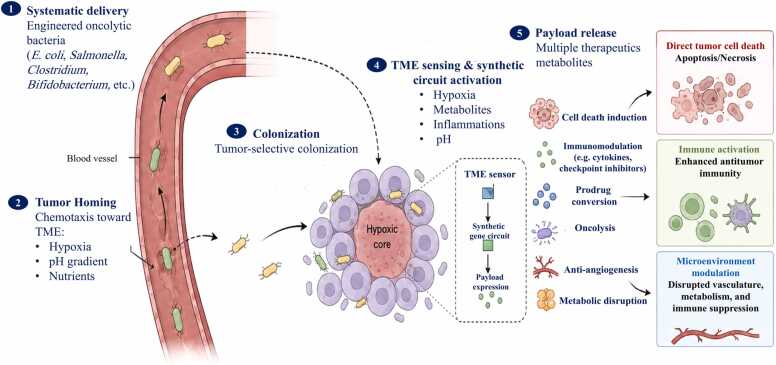
Mechanism of engineered bacterial tumor targeting mediated by synthetic gene circuits: This schematic illustration shows how therapeutic delivery is mediated by different oncolytic bacteria; they specifically localize to tumors. Tumor-specific metabolites and hypoxia are among the tumor-associated indicators that the bacteria are genetically equipped to detect. Synthetic gene circuits are activated upon accumulation in the tumor microenvironment (TME), thereby regulating the production of therapeutic payloads, such as prodrug-converting enzymes, cytotoxins, or immunomodulatory agents. This regionally constrained stimulation enhances therapeutic accuracy while limiting off-target damage, raising the overall safety profile of microbial-based cancer therapy.

The fundamental advantage of bacteria-based cancer therapy is the capability to specifically target tumors via unique mechanisms [28]. Bacterial colonization and growth are facilitated by the biochemical and physiological cues found within the TME (Fig. 2) [29]. TME is a heterogeneous network of cellular and acellular elements that together sustain tumor growth, progression, and dissemination by continuously providing nutrients, while simultaneously limiting effective drug delivery and impairing immune surveillance [30]. Availability of chemical attractants such as nutrition in the TME promote bacterial proliferation. Tumors create and release metabolites such as lactate, different amino acids, and sugar compounds, which act as nourishment for bacteria [31]. Additionally, the TME releases a variety of chemotactic signals that drive bacteria into the TME, including chemokines and growth factors including transforming growth factor-β and vascular endothelial growth factor [32]. Some bacteria species rely on quorum sensing to control their population dynamics and behavior within the tumor. Surprisingly, certain tumours can imitate these signals by attracting bacterial agents to colonize [33].

The TME's transformed immunological landscape also contributes significantly to the growing bacterial community within tumors. The intra-tumoral environment is frequently immunosuppressive, characterized by tumor-associated macrophages, myeloid-derived suppressor cells (MDSCs), and regulatory T cells (Tregs), reducing the effectiveness of the immune response against both the tumor and invading bacteria during colonization [34]. This milieu permits bacteria to escape and hide from immune surveillance and elimination more easily than they would in healthy tissues. Oncolytic bacteria passively penetrate the tumor tissue and become caught in the tumor's disorganized circulatory system, causing inflammation [35]. For example, results show that in the early phases of *Salmonella typhimurium* tumor colonization, tumor necrosis factor alpha (TNF-α) causes vascular disruption, which allows bacteria to enter the tumor through the bloodstream. According to studies, TNF-α levels increased significantly with bacterial intratumoral colonisation and peaked 120 min after injection. More vessel disruption results from this increase, which creates the ideal environment for bacteria to grow [36]. Another example are *Listeria* species when selectively target malignant cells by infecting them intracellularly. *Listeria* not only infiltrates the TME but also evades the host's immune system by infecting myeloid-derived suppressor cells (MDSCs) [37], [38]. In contrast, the TME significantly decreases the immunological response, preventing the elimination of pathogens. This method then transfers them from Myeloid-Derived Suppressor Cells (MDSCs) to cancer cells through a unique cell-cell dissemination pathway. Moreover, immune-stimulating MDSCs infected with *Listeria* produce the cytokine IL−12, which increases T and NK cell responses [37], [38].

Leaky vessels also exhibit an enhanced permeability and retention (EPR) effect [39]. The enhanced permeability and retention (EPR) effect typically arises from structural and functional alterations in tumors, including increased extracellular matrix (ECM) stiffness, reduced lymphatic drainage, vascular hyperpermeability, and elevated interstitial fluid pressure (IFP) [40]. However, EPR-based targeting relies on assumptions that may not hold in practice. These hypotheses comprise unidirectional mass transfer to cancer cells, independent of concentration gradients; variations in interstitial fluid pressure (IFP) caused by vascular leakage; transport resistance; and the selective influence of IFP on lymphatic vessels compared to blood vessels [41].

Beyond passive accumulation, the hypoxic tumor microenvironment (TME) presents a critical target for selective microbial colonization. One of the most important factors attracting facultative/obligate anaerobes is hypoxia which is recognized as an essential hallmark of the TME in virtually all solid malignancies, creating a unique physiological landscape that microorganisms can exploit for targeted colonization [42]. Most cancers have low oxygen levels at their cores as a result of tumor cell proliferation, which typically outpaces angiogenesis. Bacterial pathogens such as *Clostridium, Salmonella,* and *Bifidobacterium* species thrive in these low oxygen environments [43]. Conventional immunotherapeutic approaches often fail to account for the immunosuppressive effects of tumor hypoxia (TME) [44]. Consequently, recombinant anaerobic bacteria are being developed to counteract these tumor niches. Given its essential role in regulating disease progression and mediating immune evasion, the hypoxic TME serves as an ideal physiological trigger for combination microbial therapies, allowing for localized activity that is largely absent in healthy, oxygenated tissues (B. Wang et al., 2021).

Toxin-based targeting also had been reported when studies revealed exotoxins from *Clostridium* species can disrupt cancer cell membranes, inhibit important biological activities, and eliminate tumors [45], [46], another study showed anticancer activity of shiga-like toxin from *E. coli* 157 in *in vitro* model [47]. Moreover, tumors infected with *Clostridium* cause granulocytes, macrophages, and CTLs to migrate to the infection site, which promotes cancer regression [48]. Due to immune cell movement, neutrophils release tumor necrosis factor (TNF)-related apoptosis-inducing ligand (TRAIL), which is a novel mechanism of anticancer activity [49].

Moving beyond metabolic sensing and bacterial toxins, microbial cell-surface display systems offer an effective platform for this aim by enabling the display of peptides and proteins on microbial cell surfaces through genetic fusion to anchoring motifs. These anchoring motifs naturally contain native cell-surface proteins or their functional domains, which act as carriers to ensure stable surface localization [50]. This approach supports more precise molecular tumor diagnosis and enables the development of “smart bullet” therapeutics that are both more effective and less toxic [51]. For example, Chang *et al.* proved that *Escherichia coli* selectively targeted HER2-positive cancer cells *in vitro* when engineered to show an anti-HER2/neu affibody on its surface [52]. In a separate study, Massa *et al.* treated mice bearing human lymphoma by attenuated *Salmonella typhimurium* expressing an anti-CD20 single-domain antibody and carrying a prodrug-converting enzyme [53]. While former studies were largely confined to hematologic malignancies or in vitro solid tumor models, the therapeutic potential of these genetically modified bacteria warrants comprehensive assessment across diverse *in vivo* systems (Park et al., 2016).

## 4 Synthetic control system for safety and precision

When employing certain bacteria against cancer, it is critical to reduce their virulence against the host immune system, keeping in mind that the inherent anticancer activity of some bacteria is attributable to their virulence factors [54], [55]. Therefore, attenuating microorganisms shouldn't result in the loss of their anticancer activity.

Genetic editing of bacterial strains is commonly used to generate attenuated oncolytic microbe or bacterial-derived oncolytic agents. For example, one of the most widely used genetic editing approach involves editing lipid A biosynthesis to reduce endotoxin toxicity in outer-membrane vesicles (OMVs) which are nanocarriers used for antitumor payload delivery and immunomodulation techniques [56]. Deleting the msbB gene by genetic editing, which produces a lipid A acyltransferase in *Salmonella typhimurium*, leads in underacylated lipid A with significantly reduced ability of microbes to trigger systematic sepsis shock. Thus, attenuation retain the antigenic complexity but demonstrate improved safety profiles [56].

Another attenuated *Salmonella* strain was created by decreasing the expression of endotoxin-associated genes or suppressing their functional activity. *Salmonella* relA- and spoT-mutant strains that lack the ability to synthesize ppGpp, a signaling molecule involved in toxin gene production, showed low toxicity. The LD50 value of the ΔppGpp strain was up to 10^5^−10^6^-fold higher than that of wild-type strains [57]. Deletion of the phoP and phoQ genes which encode a critical two-component system that acts as a major virulence regulator did not impair *Salmonella's* anticancer activity, whereas the deletions lowered its virulence in normal tissue [58]. Strains with these mutations have been utilized to make an excellent vaccine, and have recently been exploited as a delivery vehicle for tumor therapies [59]; [60], [61].

*L. monocytogenes* cytotoxicity is caused by the deletion of genes involved in cell invasion and phagolysosome release, which is accomplished via HIy deletion [62]. Mutant strains of *L. monocytogenes* lacking inIA and inIB are unable to invade, while strains lacking ActA or ActA PESTf-like sequences lack intracellular diffusion capacity [63].

In the development of bacterial delivery systems, genetic engineering plays a vital role, particularly by increasing bacterial targeting ability to tumors and reducing their toxicity. Genetic engineering tactics use genetic tools such as plasmids, phages, gene circuit and gene editing technologies like CRISPR-Cas9 to manipulate the bacterial genome.

**Fig. 3:**
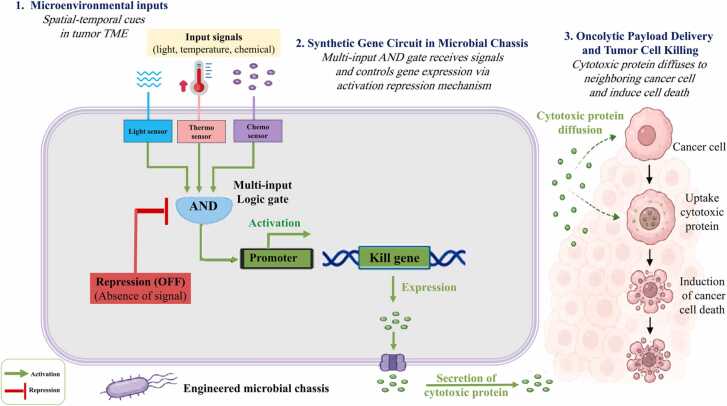
Diagram of a Synthetic Gene Circuit for Targeted Oncolytic Payload Delivery: The diagram illustrates a synthetic gene circuit engineered into the microbial chassis. A multi-input logic gate (e.g., an AND gate) integrates specific microenvironmental signals (such as temperature, light, or chemical cues). Gene expression is tightly controlled by both activation (green arrow) and repression (red T-bar) mechanisms. Upon activation, the bacterial promoter drives the expression and secretion of a cytotoxic protein, which subsequently targets neighboring cancer cells, and inducing cancer cell death.

Advancements in synthetic biology have enabled bacteria to sense and respond to specific stimuli through the use of genetic circuits [64]. A genetic circuit consists of interlinked biological elements that encode polypeptides or RNA molecules. These elements enable single cells to recognize, analyze, and respond to environmental or intercellular signals to carry out specific logical functions (Fig. 2, Fig. 3) [65]. The suicide switch is one of the genetic circuit mechanisms that produces programmed autolysis of bacteria under specified conditions [66]. In this regard, a modified *Salmonella* strain driven by the PsseJ promoter, which is triggered when the bacteria are in the intracellular environment, causing the development of the lytic enzyme LysE, resulting in spontaneous bacterial lysis [67]. This lytic mechanism allows bacteria to release therapeutic proteins after penetrating tumor cells, resulting in targeted cancer cell treatment (Y. [64]). Another approach used different suicide switch induction when the suicide gene circuit induced by physical conditions rather than chemical inducers to avoid the high cost of chemical inducers, potential toxicity, and the risk of target gene leakage. For instance, a modified strain of *Escherichia coli* called EcN-pDawn-φx174E/TRAIL was developed. This strain lyses when exposed to blue light and releases the tumor apoptosis-inducing ligand TRAIL, allowing for light-controlled cancer therapy ( [68]; Y. [69]).

Detecting complex environmental conditions, often characterized by the concurrent presence of multiple physicochemical signals (e.g., pH, temperature, small molecules, or metal ions), requires multi-input genetic logic gates. Such architectures markedly improve sensing specificity and accuracy in biological regulation. Additionally, these logic gates are engineered to detect cancer-specific markers, for instance AND-gate (Fig. 3) can be programmed to require simultaneous presence of two markers to restrict activation to cells that co-express both markers, preventing off-target effects in healthy tissue [70]. Another example is a prominent application involves genetic circuits engineered to activate tumor-suppressor responses, such as p53 activation, only when specific environmental stresses, such as hypoxia and radiation, are recognized simultaneously [71]. This dual-input functions as a safety switch, preventing accidental activation in healthy cells.

## 5 Comparative analysis of microbial chassis

Tumors have been found to harbor *E. coli*, *S. typhimurium, Clostridium* species, *Bifidobacterium* species, *Vibrio cholera*, *Shigella* species and *L. monocytogenes*; some of these have been used to treat tumors using wild type or gene-modified strains [72], [73], [74], [75], [76]. Oncolytic therapy can be accomplished by their toxins, enzymes, biofilms, and other secondary metabolites, as well as their spores, which leads to direct or indirect destruction of cancer cells [77].

*E. coli* and *Salmonella* species are facultative anaerobes which have the advantage of grow in both oxygenated and hypoxic niche that makes them versatile. For designed oncolytic therapy, *E. coli* is superior to *Salmonella* due to its ease of programming for accurate tumor targeting and therapeutic delivery (J. [78]). Furthermore, the whole-genome sequencing of *E. coli* has also facilitated precise genetic manipulation through synthetic biology approaches [79].Non-pathogenic strains, like Nissle 1917, are valued for their safety, capacity to colonize solid tumors with minimal oxygen, and genetic tractability (J. [78]). This strain has been engineered to express pH (low) insertion peptide (pHLIP), which can rapidly transport antitumor drugs. *E. coli* strain K−12 is also employed in cancer treatment. It is genetically engineered to generate the hemolytic protein cytolysin A (ClyA). It induces apoptosis by pore-forming action, hence inhibiting tumor growth [80], [81]. Also, toxin-mediated antitumor mechanism was recorded when shiga-like toxin I from enterohemorrhagic *E. coli* O157 exhibited anticancer activity when mixed with probiotic supernatants against the Caco−2 cell line by inducing necrosis [47]. Over-expression of metabolite is another anticancer mechanism when genetically engineered *E. coli* strains were designed to overexpress catalase, allowing the decomposition of H₂O₂ within solid tumors [82].

*Salmonella* spp. on the other hand, is known for its superior motility and "tumor-homing" characteristics, resulting in higher concentrations (typically 10^^8^ CFU/g) in tumors (C. Z. [83]). Also, it has higher persistency as it can reside longer in hypoxic/necrotic area [7]. Additionally, *Salmonella* spp. especially *S. Typhimurium* often more efficient in accumulation in deep necrotic/hypoxic tumor tissues than normal tissues (>1000:1) [84].

Tumor destruction can be achieved by injecting anaerobic bacteria or their spores directly into the tumor or systemically. These bacteria develop specifically in hypoxic locations. Crucially, treatments based on anaerobic bacteria like *Clostridium* spp*.* and *Bifidobacterium* may be able to bypass many of the drawbacks of systemic treatments and provide a precise means of eliminating cancers that would otherwise be incurable. They preferentially colonize and multiply in hypoxic areas of the body because of their obligatory anaerobic nature, which makes them a perfect option for targeting solid tumors [2], [46]. While the bacterial spread of facultative anaerobes like *E. coli* and *Salmonella* might not be limited to tumor but can be damage healthy tissues as recorded with *Salmonella typhimurium* VNP20009 [85], obligate anaerobes provide a natural containment because they physically cannot survive outside the tumor's dead necrotic center. Which boost their safety and selectivity in tumor-targeting strategy [86].

*Clostridium* novyi-non-toxic (NT) is at the lead of tumor necrosis therapy, showing promise in preclinical models [87]. The most superior strain was found to be *Clostridium novyi* (C. novyi, ATCC #19402), which was then made nonpathogenic by removing a residential phage that carried α-toxin, a key toxin that causes toxicity [87].

It spreads throughout the tumor and slows its growth by inducing necrosis and cell lysis. It has successfully completed phase I trial which tested the safety and tolerability of a single intratumoral injection of *Clostridium*-NT spores in patients with advanced solid tumors, and II clinical studies. *C. novyi*-NT increases inflammatory responses by interacting with pro-inflammatory cytokines such as IL−6, MIP−2, TIMP−1, and G-CSF [88], [89]. These cytokines improve adaptive anti-tumor immunity by attracting immune cells to the site of infection. Additionally, *C. novyi*-NT can penetrate the hypoxic region of tumors that are resistant to other treatments such as radiation and chemotherapy. As a result, it is commonly used in conjunction with radiation therapy or chemotherapy medications. This method is known as Combination Bacteriolytic Therapy (COBALT) [7].

Another approach to employing *Clostridium* has been recoded as a tumor-specific gene delivery vector, mainly in the context of *Clostridium*-directed enzyme prodrug treatment (CDEPT). CDEPT is a cancer gene therapy strategy that uses an exogenous therapeutic enzyme produced by *Clostridium* to convert normally inert prodrugs into cytotoxic, active metabolites. This kind of localized prodrug activation can, in theory, result in constant active drug concentrations within the tumor microenvironment and improve the therapeutic index [87]. Cytotoxic compounds may diffuse into and kill nearby cells inside the tumour, depending on the tissue penetration capacity of the active metabolite(s). This is known as the 'bystander effect'. A strong bystander effect would allow cell killing to occur outside of the necrotic borders, where *Clostridium* germination is possible [87].

Although *Bifidobacterium* and *Clostridium* both preferentially colonize and multiply in hypoxic areas of the body, *Bifidobacterium* are non-pathogenic probiotics that are present in the human gut, in contrast to *Clostridium*, which is potentially harmful [90]. This makes them the safer choice; even if they were to escape the tumor, they are generally recognized as safe (GRAS) by the body. However, due to their inherent biosafety, they do not naturally kill tumor cells when compared to *Salmonella* or *Clostridia*. As a result, they are mostly serving as a delivery vehicle for therapeutic agents and a valuable tool for enhancing the effectiveness of traditional treatment methods such as chemotherapy, radiotherapy, and immunotherapy [2].

Internalizing into the host cytosol and release antitumor drug is another strategy that microbial chassis can employ. While extracellular colonization is useful for localized medication release, *Listeria monocytogenes* and *Shigella* belong to a distinct family of intracellular vehicles. *Listeria monocytogenes* is the most commonly used strain for cancer treatment. As a facultative anaerobic intracellular bacterium, it lives and replicates in the cytoplasm of the host cell [7], [77]. It can escape the phagolysosome and transfer plasmid DNA into the cytoplasm. It has been programmed to express tumor-associated antigens (TAAs) like melanoma-associated antigen-B (MAGE-B) (S. H. [91]). It also produces a pore-forming protein known as listeriolysin O (LLO). LLO generates pores in endosome membranes and helps deliver DNA molecules into the cell's cytoplasm [92], [93]. This distinguishing feature makes it an efficient carrier for anticancer drugs and it is noteworthy that engineered *L. monocytogenes* had made it through phase I, II, and III in clinical trials [7].

Similarly, *Shigella* has a type 3 secretion system capable of administering toxic proteins into the host cell cytosol. Furthermore, *Shigella* has a limitless cassette space to encode therapeutic proteins. Studies have shown that engineered *S. flexneri* can be adapted to preferentially internalize into glioblastoma cells, with one specialized strain showing a 124-fold higher affinity for glioblastoma cells compared to normal tissue [75].

In summary, selecting a microbial chassis needs a balance between targeted precision and clinical safety. While *Salmonella* and *E. coli* provide superior engineering modularity, obligate anaerobes such as *Clostridium* and *Bifidobacteria* provide inherent safety via necrotic niche restriction. Additionally, the inclusion of *Listeria* and *C. novyi*-NT in clinical studies (Phase I/II) demonstrates the progress of these stages from experimental designs to practical oncological therapies.

## 6 Cytotoxic payloads and killing strategies

Various killing strategies have been recorded in bacterial-based anticancer therapy. Direct cancer cell lysis is one of the strategies employed [94]. Oncolytic bacteria can invade cancer-bearing tissue directly (Fig. 1), releasing toxins that damage the functioning of the attacked cells as well as enzymes that lyse them. For example, *Clostridium novyi* -NT produces alpha-toxin, which can damage and destroy cell membranes, resulting in cell rupture and death. Another method employs the *S. typhimurium* bacterium, which synthesizes bacterial proteins and induces tumor cell apoptosis [95]. Furthermore, oncolytic bacteria can destroy cancer cell by nutrient depletion [96]. The anoxic area, or deoxygenated tumor tissues, encourages the growth of obligatory anaerobic bacteria [97]. Reduced nutrient and oxygen supply causes the necrotic areas to form, which in turn causes the viable blood vessels to break down and the tumor cells to die from starvation [36].

In addition to cell lysis and cancer starvation strategies, immune response induction is another critical killing strategy employed by oncolytic bacteria. These bacteria can directly attack and damage cancer cells, causing the release of tumor-associated antigens and inducing a stronger immune response from the host. This strategy not only helps to reduce tumor mass, but it also aids to generate long-term anti-tumor immunity by boosting both innate and adaptive immunity. As a result, the body's immune system is prepared to target the remaining cancer cells, perhaps reducing the risk of recurrence [98]. Also, the continuous production of tumor-associated antigens into the tumor's extracellular fluid enhances dendritic cell (DC) immigration and activation. These DCs subsequently internalize, process, and present antigens to T cells, resulting in a unique adaptive immunological response. This series of events affects the progression of metastatic cancer cells as well as the primary tumor itself [99].

Alternative strategy involves bacterial toxins and immunotoxin which have been deeply researched as possible anti-cancer therapies ( [100], [101], [102]; S. [103], [104]). There are numerous reasons why the use of bacterial toxins as a cancer treatment could be beneficial. A primary benefit is potency; bacterial toxins are generally effective in small doses, implying minor systemic toxicity [105]. Another advantage is that scientists could improve the specificity of targeting cancer cells by employing genetic engineering to create fusion proteins that incorporate specific toxins, commonly in the form of antibodies or growth factors. This method would avoid unexpected injury to normal tissues. For example, immunotoxins (antibodies fused with toxins) have demonstrated potential efficacy for a spectrum of cancers, hematological malignancies, and solid tumors in preclinical investigations and may give a pathway forward to market [102].

The clustered regularly interspaced short palindromic repeat-associated protein 9 (CRISPR-Cas9) genome editing technology has revolutionized the ability to produce several recombinant oncolytic strains like *Clostridium*. CRISPR-Cas9 enables the addition, modification, or deletion of genetic material at specific sites in the genome by directing the Cas nuclease to its target via short RNA 'guide' sequences. To ensure a long-lasting and effective killing strategy, recent breakthroughs have used CRISPR/Cas9 nickase (Cas9n) to permanently arm *Clostridium novyi*-NT. This technique uses the strain's natural homology-directed repair (HDR) to accomplish stable, chromosomal integration of therapeutic payloads by creating single-stranded DNA nicks rather than the fatal double-strand breaks found in regular Cas9 [106]. By avoiding the toxicity of endogenous CRISPR "immune" responses, this synthetic biology method ensures that, once spores germinate, the resulting vegetative cells serve as permanent, enclosed factories for the constant and lethal release of cytotoxic payload within the tumor core.

**Fig. 4:**
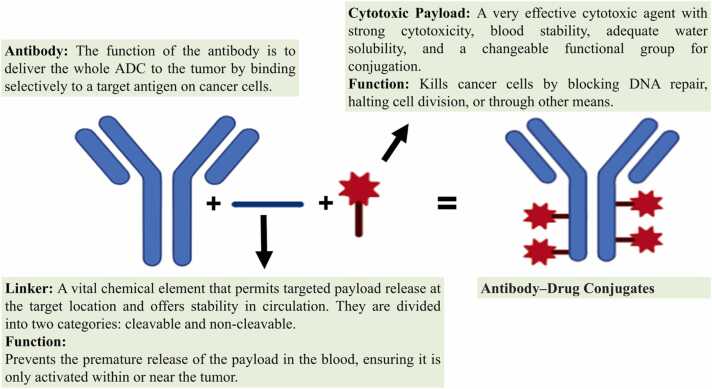
Structure of Antibody-Drug Conjugate (ADC): The three main parts of an ADC are antibodies, linkers, and cytotoxic payloads.

Another killing strategy is the use of bacterial toxins in antibody-drug conjugates (ADCs) takes use of their ability to disrupt critical cellular processes, resulting in rapid and permanent cell death [107]. ADCs are targeted therapeutic agents composed of highly defined monoclonal antibodies chemically related to potent cytotoxic small molecules [108]. An ADC consists of three important components: an antibody, a cytotoxic payload, and a linker that joins the two (Fig. 4). After administration, the antibody moiety selectively fixes to its corresponding antigen expressed on the surface of tumor cells, leading to internalization of the ADC–antigen complex. Other ADC–antigen complexes are transferred to lysosomes, where enzymatic degradation or the acidic microenvironment cleaves the linker, resulting in the cytotoxic payload release. The liberated cytotoxic agents then exert their effects by inducing DNA damage or inhibiting cell division, ultimately causing tumor cell death [109].

In addition to delivering a cytotoxic payload by ADCs, certain payloads show a “bystander effect,” whereby the intracellularly released drug diffuses out of the targeted tumor cell, enters neighbouring antigen-negative cells, and induces cell death [110]. The range to which bystander payloads penetrate tumor tissue inside the tumor microenvironment remains poorly characterized. Moreover, it is unclear whether the diffusion of released payloads can sufficiently compensate for heterogeneous antibody distribution. Although bystander effects are generally thought to increase ADC efficacy, present techniques for assessing bystander effects' cell killing efficiency offer insufficient or partial resolution for insights that can be applied in a clinical setting [111]. The dual nature of ADCs is displayed by the bystander effect, which improves intratumoral payload distribution while raising the possibility of off-target damage [112].

Bacterial-directed enzyme prodrug therapy (BDEPT) is another distinct killing strategy employed by synthetic biology against cancerous cells. This strategy involves using an inactive compound that is toxic only when given as a drug and accumulates at the tumor site without affecting the rest of the body (Fig. 1). The technique begins with the prodrug being delivered systemically, and then bacterial enzymes are localized to the tumor location using either vectors (bacterial) or purified enzymes. These enzymes subsequently activate the prodrug within the TME, causing the selective death of tumor cells without damaging normal cells [113], [114]. In order to transport the enzyme to the tumor site, bacteria are "armed" with a gene that codes for a prodrug-converting enzyme that either has no human homologue or has more favorable enzyme kinetics than any human isozyme. BDEPT is a two-phase treatment, the patient is given the armed vector in the first phase, which specifically targets the tumor spot where the enzyme is expressed. The second stage involves administering a prodrug, which the expressed enzyme exclusively at the tumor site converts to a cytotoxic drug once the levels of enzyme expression are ideal. This leads to tumor-selective cytotoxicity [113].

In BDEPT and other prodrug conversion techniques, the drug is produced *in situ* after tumor targeting. This provides a variety of advantages over conventional therapy. First, tumor selectivity is accomplished since the prodrug is only converted within the tumor, limiting adverse effects in other organs. Second, an amplifying effect is obtained because a single therapeutic enzyme molecule can activate several prodrug molecules. Third, the bystander effect is achieved [113].

Beside engineered payloads, naturally derived bacterial components—such as metabolites, biosurfactants, biofilms, and spores—have been proven to act as highly effective intrinsic killing strategies in oncolytic therapies ([115], [116], [117]; L. [118]). For instance, the anticancer potential of biosurfactants is attributed to their ability to inhibit malignant cell proliferation, induce apoptosis, trigger targeted necrosis, and arrest the cell cycle. In a different approach, the therapeutic efficacy of bacterial spores relies on spatial localization; their activity is based on their capacity to germinate and proliferate in the hypoxic tumor core [95].

Ultimately these various killing strategies—synthetically engineered payloads or exploiting inherent microbial mechanisms—turn bacteria into complex, broad anticancer tools. Nevertheless, putting these powerful mechanisms into clinical practice necessitates overcoming substantial biological and regulatory obstacles.

## 7 Clinical translation and failure mode

Despite promising preclinical results, the clinical translation of microbial-based cancer therapies has been inhibited by host immune responses, safety considerations, and regulatory challenges. This gap between preclinical success and clinical efficiency was mainly evident in the Phase I trial of *Salmonella typhimurium* VNP20009, which revealed preferential tumor colonization but did not achieve sufficient therapeutic advantage [119]. The defense mechanisms of the host play an important part in this limitation by quickly identifying and destroying modified microbes, limiting their persistence, intratumoral spread, and successful payload delivery. Furthermore, the doses that can be administered to patients are constrained by safety worries arising from systemic exposure to bacterial components and expressed toxins. Even after bacteria successfully inhabit tumors, their therapeutic efficiency may be compromised by genetic instability. Kill-switch systems are mainly susceptible to this instability, as they incorporate toxic genes that can exert deleterious effects even in the OFF state due to leaky expression. This residual toxicity creates selective pressure for loss-of-function mutants, which may appear and ultimately dominate during serial passaging [120]. In CRISPR/Cas systems, multiplex genome editing efficiency is still relatively low, and off-target effects may happen. Furthermore, the cytotoxicity related to Cas9 limits its broader application in synthetic biology and industrial cell workshops. Consequently, when adapting CRISPR/Cas to novel types, it is vital to ensure high editing efficiency while minimizing cellular toxicity and unintended off-target effects (W. [121]). Biosafety and regulatory frameworks are directly impacted by these biological and engineering uncertainty. Fears of surrounding synthetic biology encompass both biosafety dangers—such as unintentional release—and biosecurity threats, including deliberate misuse. Social sciences scholars have highlighted potential hazards, comprising horizontal gene transfer, disruption of local ecosystems and biodiversity, unintended interaction with engineered organisms, in addition to broader ethical, legal, and societal considerations [122]. One of the serious risks related to engineered living systems is their inherent unpredictability. Though some challenges must be acknowledged, including the complexity of genetic circuits, the scale of engineered metabolic pathways, thermodynamic constraints on biochemical production within cellular surroundings, and evolutionary constraints. Though many strategies exist to mitigate the detrimental effects of evolution on engineered designs [123]. Beyond safety considerations, ethical issues linked to the modification of existing types or the complete engineering of new life forms must also be taken into account [124]. Though no important biosecurity or biosafety incidents have been reported in synthetic biology to date, governance and regulatory frameworks must be taken into account by nations, individual researchers, institutions, and the global community to avoid potential future risks [125]. Synthetic biology is a growing field offering many hopeful research opportunities. To improve the efficacy of future investigations, it is important to optimize methodologies while addressing and mitigating the risks and challenges related with emerging approaches [126]. Taken together, these elements help clarify why frequent microbial therapies exhibit high efficacy in tightly controlled preclinical models yet perform suboptimally in human patients. Consequently, addressing immune clearance, genetic instability, and regulatory challenges will be crucial for the future clinical success of customized microbial cancer therapies.

## 8 Future engineering strategies

The multidisciplinary science of synthetic biology is expanding quickly and has many uses in many different industries. It has brought a revolutionary aspect to the biological sciences, arising from the fusion of engineering, physics, mathematics, and chemistry (Safaei et al., 2020). Synthetic biology is changing cancer therapy by enabling the development of modified microbes as cutting-edge living therapeutics. These tools have a number of benefits over traditional therapies, including better precision and fewer side effects. Genetic circuits can prevent bacterial development in aerobic environments to improve natural targeting abilities, limiting the spread of cancer and protecting healthy tissues [127]. The successful transfer of bacterial therapeutics from bench to bedside in the future will require a deeper understanding of the mechanism of action, the development of comprehensive regulatory frameworks, and the integration of numerous technological platforms. Understanding the molecular foundations of bacterial treatment of cancer is crucial to logically optimizing safety and efficacy [128].

Improving bacterial tumor selectivity by genetic engineering is one key area. Tumor-specific ligands or single-domain antibodies that target antigens linked to tumors can be produced by genetically modified bacteria. A strain of *Salmonella* has recently been altered to express the integrin-binding peptide Arg-Gly-Asp (RGD) [23]. It has exhibited increased anticancer activity and tumor selectivity. Bacteria can also be programmed to express therapeutic proteins and reporter genes. This approach will be useful in cancer treatment since it allows for the synthesis of proteins that trigger cancer cells to die. Other advances in synthetic biology have allowed for the development of more precise techniques for designing *Clostridium* for selective targeting. These precise strategies for targeting hypoxic tumors have made increased immune response and other combination therapies, such as prodrugs and other therapeutic delivery, possible. The ClosTron approach, which adapted the TargeTron platform, has made it easier to insert site-specific genes into *Clostridium* [129]. This technique is based on the retrohoming of group II introns, which enables precise modification of up to 0.4 kb of DNA through targeted insertion and the use of a selective marker [130]. This genetic technique has also been used to secrete human atrial natriuretic peptide (ANP), a peptide that can inhibit inflammatory responses. Upon injection into tumors in mice, *C. novyi*-NT-ANP revealed the ability to minimize inflammation-induced mortality and increase tumor clearance [131].

Adding genetically encoded circuits that limit bacterial activation or proliferation to inducible gene circuits improves precision even more. Therapeutic payloads can be tailored to the specific needs of different tumor contexts: for "hot" tumors, bacteria deliver immunomodulators (e.g., cytokines, tumor antigens) to enhance antitumor immunity; for "cold" tumors, they express direct-killing effectors (e.g., toxins, apoptosis proteins) to compensate for poor immune infiltration [22]. Therapeutic release can be molecularly linked to tumor-specific signals in both space and time by creating tumor-specific pathogenic signal-triggered systems, allowing bacteria to adjust to the development of tumors. Logic-gate designs have the potential to improve tumor treatment precision. For instance, *E. coli* Nissle 1917 has been designed to release hemolysin under TME conditions of hypoxia, low pH, and high lactate levels, utilizing an XOR amplifier to target colorectal cancer (T. [132]). In a similar way an AND logic gate was built in *E. coli* Nissle 1917, ensuring therapeutic protein release only in the presence of both lactate and acyl homoserine lactone **(**AHL), thus limiting leakage [133].

Furthermore, modified live oncolytic bacteria paired with other anticancer techniques such as nanomaterials and oncolytic virus therapy could improve therapeutic coverage, overcome monotherapy limits, and increase tumor-killing capacity. Future research should focus on combining oncolytic bacterial therapeutics with existing modalities, such as checkpoint inhibitors or standard chemotherapies, to inform combinatorial methods that improve immune responses to tumors. Using bacteria to improve the administration of immune checkpoint inhibitors, resulting in higher antitumor immunity. Additionally, bacterial compounds can be employed to modify the TME, improving immune infiltration and function [29].

The combination of personalized medicine for cancer therapy with synthetic biology to develop oncolytic bacteria has the potential to transform therapeutic approaches. Personalized medicine can improve efficacy by selecting medicines based on the tumor's genetic, molecular, and cellular characteristics while reducing side effects. Genetic variations influence an individual's medication metabolism; therefore, an optimal oncolytic bacterial dose could assist in adaptive and responsive treatment. For example, with the development of live biotherapeutics (LBSs), *Clostridium* can be used in a way that is unique to each type of cancer and immune system [134].

**Table 1:** Microbial-Based Cancer Therapeutics: Synthetic Strategies and Mechanisms of Action.

**Microorganism**	**Synthetic Engineering Method**	**Mechanism of Immune Activation/Cytotoxicity**	**Safety/Spatiotemporal Controls**	**Reference**
*E. coli* Nissle	Heat-inducible switch (ultrasound-controlled), secretes checkpoint inhibitor nanobodies on heat	Immune checkpoint inhibition (anti-PD-L1), immune activation	Circuit screening with high throughput, spatial/temporal thermo-inducible	[135]
*E. coli* Nissle	A quorum regulates synchronous lysis and release; anti-PD-L1 and anti-CD47 nanobodies	Blocking local immunological checkpoints, reversing tumor immunosuppression, and increasing T-cell death	Surface functionalisation using nanobody/MOF, quorum-controlled lysis, and NIR-controlled lysis	[136], [137]
*Salmonella* *and E. coli* (chemically modified)	Checkpoint inhibitor display, thermal/photo-inducible ClyA, and engineered expression of photothermal proteins (such as melanin)	Checkpoint inhibitor-based local immunotherapy, photothermal ablation, and direct cytolysis (pore-forming)	Expression of genes induced by light or heat, external photothermal regulation	[138], [139], [140]
*Salmonella* Gallinarum (attenuated ΔppGpp)	TGFα-PE38 constitutive strong promoter (*Pseudomonas* exotoxin fusion)	Via direct cytotoxicity, a strong exotoxin is delivered to cancer cells.	Background attenuation, RES/minimal systemic expression data	[141]
*Salmonella* (AIS, htrA mutant)	Modulation of surface polysaccharides for dynamic virulence; quorum-sensing circuit for on/tumor-off/normal control	Surface engineering-based tumor-restricted cytotoxicity and immunogenicity.	Variable off-target effect, QS regulation, and controlled virulence "switch."	[142]
*E. coli* MG1655	TNF-α stable secretion in tumors by tailored designs	Localised cytotoxicity of TNF-α, pro-inflammatory tumor destruction	Tumor-homing confirmed, tested routes of delivery.	[143]
*Salmonella* Typhimurium	Surface expression of HSV-TK (prodrug-converting) and CD20 antibodies	Targeted tumor antigens, prodrug cytotoxicity triggered by GCV, and tumor lysis	Improved tumor specificity, prodrug control, and surface antibody display	[53]
*E. coli* (surface-armored with Fe-ZIF−8 NPs)	Activation of tirapazamine (from loaded NP), lactate oxidase secretion, and a hypoxia-inducible promoter	H_2_O_2_→•OH (via NP), lactate depletion, and development of local ferroptosis	NP barrier, TME-responsive activity, and regulated secretion	[144]
*E. coli* (engineered circuit)	Light-controlled switch between planktonic, biofilm, and lytic lifestyles using a programmable circuit	Effect of light-induced in situ cytolysis and transport of payload	Dosage control using a hierarchical circuit and near-infrared (NIR) light programming	[145]
*S. typhimurium VNP20009*	Physical interaction	Radio-immunotherapy	hitchhiking on CD11b + immune cells toward tumor then X-ray-induced activation	[144])
*L. monocytogenes*	Chemical bonding	Chemo-immunotherapy	natural tropism for immunosuppressed tumor sites	[146]
*L. innocua*	Physical interaction	Microbial sonosensitizer enables hypoxia-relieving sonodynamic therapy (SDT)-induce immunogenic cell death (ICD)	ultrasound radiation	[147])
*Clostridium butyricum*	Chemical bonding	Anaerobic bacteria deliver radiosensitizer-immune activator	hypoxia-driven tropism and radiotherapy-induced bacterial death	[148]
*B. bifidum*	Physical interaction, Chemical bonding	Probiotic-driven dual-mode photodynamic therapy (PDT)/SDTROS bomb with DR5 targeting	non-pathogenic probiotics naturally target hypoxic tumor regions	[121]
*L. lactis*	Gene editing	Probiotic-delivered Flt3L-OX40L reprograms Dendritic cells (DC-T-cell) axis	localized intratumoral administration limiting systemic exposure	[149]

## 9 Conclusions

Microbial cancer therapy has evolved from a conceptual framework to a rapidly expanding therapeutic infrastructure, primarily due to advancements in synthetic biology. This discipline has enabled the creation of programmable devices with higher safety profiles, controlled cytotoxic activity, and specific tumor-targeting capacities. Nowadays, engineered bacteria are able to transport prodrugs or cytotoxic chemicals, recognize signals unique to tumors, activate synthetic gene circuits, and govern the immune microenvironment. Additionally, advances in genome editing, kill-switch mechanisms, and surface exhibition methods have greatly increased the biosafety, management, and adaptability of microbial-based treatments. Notwithstanding these developments, there are still significant obstacles to overcome, such as genetic instability, off-target toxicity, insufficient tumor colonization, immune-mediated clearance, manufacturing constraints, and regulatory restrictions. A number of these limitations result from the disconnected optimization of key elements rather than their coordinated development within an integrated therapeutic framework, including microbial chassis selection, genetic circuit architecture, payload control, and biosafety procedures.

More reliable and stable synthetic circuits, better spatiotemporal modulation of therapeutic action, uniform biosafety measures, and a more thorough integration of computational modeling and rational design techniques are all necessary to address these problems.

In summary, tailored cytotoxic microorganisms are a promising next-generation therapeutic approach in oncology that may supplement or perhaps outperform traditional therapy strategies. However, persistent innovation in synthetic biology, together with rigorous preclinical validation and well-designed clinical trials, will be required to convert these technologies into reliable, efficient, and therapeutically applicable anticancer therapies.

## CRediT authorship contribution statement

**Nawfal Haitham Al Shaikhli:** Writing – review & editing, Writing – original draft, Data curation. **Mustafa Attiyah Hadid:** Writing – review & editing, Writing – original draft, Visualization, Supervision, Software, Project administration, Formal analysis, Data curation, Conceptualization. **Challoob Muhtada Ali:** Writing – review & editing, Data curation. **Samar Thamer Hameed:** Writing – review & editing, Visualization, Data curation. **Omar Khalid Suhail:** Writing – review & editing, Visualization, Software, Formal analysis.

## Declaration of Generative AI and AI-assisted technologies in the manuscript preparation process

During the preparation of this work the author(s) used Gemini Pro in order to improve language readability. After using this tool, the author(s) reviewed and edited the content as needed and take(s) full responsibility for the content of the published article.

## Funding

This research received no specific grant from any funding agency in the public, commercial, or not-for-profit sectors.

## Disclosure of Interest

The Authors report there are no competing interests to declare.

## Data Availability

No data was used for the research described in the article.
